# Potency of bacterial sialidase *Clostridium perfringens* as antiviral of Newcastle disease infections using embryonated chicken egg *in ovo* model

**DOI:** 10.14202/vetworld.2022.1896-1905

**Published:** 2022-08-06

**Authors:** Ryan Septa Kurnia, Simson Tarigan, Christian Marco Hadi Nugroho, Otto Sahat Martua Silaen, Lily Natalia, Fera Ibrahim, Pratiwi Pudjilestari Sudarmono

**Affiliations:** 1 Doctoral Program in Biomedical Science, Faculty of Medicine, Universitas Indonesia, 10430, Jakarta, Indonesia; 2 Indonesian Research Centre for Veterinary Science, RE Martadinata No. 30, 16124, Bogor, West Java, Indonesia; 3 Animal Health Diagnostic Laboratory, PT. Medika Satwa Laboratory Kp. Kayumanis, 16166, Bogor, West Java, Indonesia; 4 Department of Clinical Microbiology, Faculty of Medicine, Universitas Indonesia, 10430, Jakarta, Indonesia

**Keywords:** *Clostridium perfringens*, lectin, polysaccharide, sialidase, viral replication

## Abstract

**Background and Aim:**

*Clostridium* toxins are widely used as medicinal agents. Many active metabolic enzymes, including sialidase (neuraminidase), hyaluronidase, and collagenase, contribute to the mechanism of action of these toxins. Sialidase from *Clostridium perfringens* recognizes and degrades sialic acid receptors in the host cell glycoprotein, glycolipid, and polysaccharide complexes. Sialic acid promotes the adhesion of various pathogens, including viruses, under pathological conditions. This study aimed to investigate the potential of *C. perfringens* sialidase protein to inhibit Newcastle disease virus (NDV) infection *in ovo* model.

**Materials and Methods:**

*C. perfringens* was characterized by molecular identification through polymerase chain reaction (PCR) and is cultured in a broth medium to produce sialidase. In addition, sodium dodecyl sulfate-polyacrylamide gel electrophoresis analysis was conducted to characterize the sialidase protein. In contrast, enzymatic activity and protein concentration were carried out using a neuraminidase assay kit and Bradford to obtain suitable active substances. Furthermore, embryonated chicken egg models were used to observe the toxicity of several sialidase doses. Then, the hemagglutination (HA) titer was obtained, and absolute quantitative reverse transcription–PCR assay was performed to measure the viral replication inhibitory activity of sialidase against NDV.

**Results:**

Each isolate had a specific sialidase gene and its product. The sialidase derived from *C. perfringens* could hydrolyze the sialic acid receptor Neu5Ac (2,6)-Gal higher than Neu5Ac (2,3)Gal in chicken erythrocytes, as observed by enzyme-linked lectin assay. A significant difference (p = 0.05) in the HA titer in the pre-challenge administration group at dosages of 375 mU, 187.5 mU, and 93.75 mU in the competitive inhibition experiment suggests that sialidase inhibits NDV reproduction. Quantification of infective viral copy confirmed the interference of viral replication in the pre-challenge administration group, with a significant difference (p = 0.05) at the treatment doses of 750 mU, 375 mU, and 46.87 mU.

**Conclusion:**

The potency of sialidase obtained from *C. perfringens* was shown in this study, given its ability to reduce the viral titer and copy number in allantoic fluids without adversely impacting the toxicity of the chicken embryo at different concentrations.

## Introduction

*Clostridium* is a genus of rod-shaped Gram-positive, anaerobic, and endospore-forming bacteria. These microorganisms are present in the environment and in the digestive tract of humans and animals with a relatively limited amount [1]. In addition, *Clostridium* can release the highest level of toxins as compared to other genera of bacteria. *Clostridium* toxins have diverse structures and mechanisms of action for host cells. Nevertheless, some *Clostridium* toxins have the prospect of being used as therapeutic agents [2]. Neurotoxins from *Clostridium* have been extensively studied in medical applications in the past 25 years. It has been used as a therapy for cases of muscle spasms and chronic pain and for cosmetic purposes [3, 4]. To date, research related to various other potential *Clostridium* toxins is still being studied for medical application. In addition to these toxins, other active metabolic substances and enzymes play a role in the mechanism of toxins, including sialidase (neuraminidase), hyaluronidase, and collagenase [5].

Sialidase from *Clostridium perfringens* recognizes and releases the structure of sialic acid receptors from host cells in the glycoprotein, glycolipid, and polysaccharide complexes [6]. Sialic acid mediates the attachment of various pathogens, including bacteria, toxins, viruses, protozoa, and microfungi, under pathological conditions [7]. At least eight types of viruses with various families infect host cells through sialic acid receptors, which include the Orthomyxoviridae, Paramyxoviridae, Coronaviridae, Reoviridae, Picornaviridae, Parvoviridae, and Adenoviridae viruses [8, 9]. The previous study by Burrell *et al*. [10] was conducted to inhibit viral infections by degrading receptors on the surface of respiratory mucosal cells using enzymes derived from bacteria. This enzyme is derived using a recombinant fusion protein from *Actinomyces viscosus* as an initial step in preventing viral infections through the respiratory tract. The release of the sialic acid receptor causes the failure of the virus to attach and enter the host cells *in vitro* and *in vivo*, so that virus replication does not occur [11]. In 2009, Worrall *et al*. [12] also proved that sialidase originating from *C. perfringens* type A in the intranasal vaccine mixture could protect poultry from outbreaks of the H5N1 subtype avian influenza virus. Sialidase is an initial step in preventing viral infections through the respiratory tract by degrading the sialic acid receptors of the host mucosal epithelial cells. However, the mechanism of viral replication inhibition in cells caused by sialic acid hydrolysis by sialidase from the bacterium *C. perfringens* has never been proven *in vitro*. Furthermore, the previous research was limited to hypotheses based on the concurrent administration of sialidase in intranasal vaccines.

Research on the potential of *C. perfringens* sialidase as a competitive inhibitor of viral infection *in vitro* has never been conducted. Thus, research on the purification of crude sialidase from selected isolates of *C. perfringens* bacteria and its activity needs to be carried out. Furthermore, the potential of sialidase as a competitive inhibitor in inhibiting viral replication needs to be proven.

This study aimed to investigate the potential of *C. perfringens* sialidase protein to inhibit Newcastle disease (ND) virus (NDV) infection *in ovo* model. With the *in ovo* model using embryonated chicken eggs, the activity of sialidase from *C. perfringens* would be easier to observe because it is the material commonly used for virus propagation. The findings of this study could be used as a starting point for future research on the efficacy of bacterial sialidase, which could be used as an alternative in preventing viral infections.

## Materials and Methods

### Ethical approval

The study was reviewed and approved by the Ethics Committee of the Faculty of Medicine, University of Indonesia – Cipto Mangunkusumo Hospital, in the session of December 21, 2020 (No. KET-1482/UN2.F1/ETIK/PPM.00.02/2020).

### Study period and location

This study was conducted from July 2020 to February 2022 in several laboratories including Central Laboratory of Virus and Cancer Pathobiology Research, Faculty of Medicine, Universitas Indonesia; Research and Development Unit Laboratory of PT Medika Satwa Laboratories, Bogor; National Veterinary Drug Assay Laboratory, Bogor, Indonesia.

### Identification of *C. perfringens* isolates

Bacterial isolates used as candidate seeds were archival isolates derived from the isolation and identification in the Sukabumi poultry area, West Java, Indonesia. The bacteria were stored in freeze-dried ampoules and then grown in blood agar and incubated anaerobically at 37°C for 18 h. Then, the growing colonies were observed through macroscopic and microscopic observations and extracted using the Geneaid Genomic DNA Mini Kit, following the manufacturer’s instructions [13, 14]. Molecular identification using the multiplex polymerase chain reaction (m-PCR) to characterize the genetic diversity shared by many isolates of the bacteria *C. perfringens* was performed. The amplification process used m-PCR, with a total reaction volume of 10 mL, consisting of template DNA, Master Mix KAPA2G Fast Multiplex PCR Kit (Roche, Indianapolis, USA), forward and reverse primers (Table-1) with a concentration of 10 mM, and H_2_O [15, 16].

**Table-1:** Toxin and sialidase genes and primer sequences used for multiplex PCR identification.

Type	Gene	Primer sequence	Amplicon (base pair)	Annealing temperature (°C)
*C. perfringens* toxin [15]	*cpα* (alpha)	(F) 5′-GCTAATGTTACTGCCGTTGA-3′	324	53
		(R) 5′-CCTCTGATACATCGTGTAAG-3′		
	*cpβ* (beta)	(F) 5′-GCGAATATGCTGAATCATCTA-3′	195	53
		(R) 5′-GCAGGAACATTAGTATATCTTC-3′		
	*cpε* (epsilon)	(F) 5′-TGGGAACTTCGATACAAGCA-3′	376	53
		(R) 5′-AACTGCACTATAATTTCCTTTTCC-3′		
*C. perfringens* sialidase [16]	*NanH*	(F) 5′- CTGCAATTCAAGGTGTTGGTG-3′	306	56
		(R) 5′- CTTGTCTTCTAAGCTCATATCC-3′		
	*NanI*	(F) 5′- CAAGAGTTGGTTTTGAGC-3′	467	56
		(R) 5′- AAATAAGGCTGGTATTCTG-3′		
	*NanJ*	(F) 5′- AATTGGATGGCTAGGTGGAGTT-3′	285	56
		(R) 5′- CAGGTGCTTCCTAAATCGTGAG-3′		

PCR=Polymerase chain reaction, *C. perfringens=Clostridium perfringens*

### Sialidase enzyme synthesis and purification step

The production of sialidase was performed under anaerobic conditions at 37°C overnight, and during the production process, the pH was maintained at 7. The supernatant and pellet of the bacterial cell were separated after the procedure by centrifugation at 5000× *g* for 20 min at 4°C. The pH of the supernatant was then decreased to 5 to inactivate the toxin activity, and then, the protein was precipitated with ammonium sulfate [17]. Afterward, the dialyzed residue was purified by ion exchange using Q Sepharose^®^ Fast Flow (Merck, Germany) and stepwise elution NaCl buffer. Based on the analysis of the sialidase enzymatic activity, protein concentration, and sodium dodecyl sulfate-polyacrylamide gel electrophoresis (SDS-PAGE), the fraction of sialidase protein was obtained in 0.2 M NaCl buffer elution. The final purification was performed using affinity chromatography with oxamic acid agarose [18].

### Determination of sialidase activity and protein concentration

The used isolates were initially identified by observing the sialidase activity using the receptor destroying enzyme (RDE) method, which uses the hemagglutination inhibition (HI) response and erythrocyte deposits on the bottom of the microplate [12]. Observation of sialidase enzyme activity was also conducted using the neuraminidase assay kit MAK121 (Merck) to obtain a quantitative value in U/mL. Subsequently, the concentration of the obtained sialidase protein was measured using the Bradford method [17].

### Identification of sialidase proteins

*C. perfringens* sialidase was identified using SDS-PAGE analysis. The SDS-PAGE was performed in the omniPAGE Mini Vertical Protein Electrophoresis System (Cleaver Scientific, UK). The PageRuler Plus Prestained Protein Ladder marker (Thermo Scientific, USA) compares molecular weight with a range between 10 and 250 kDa [19].

### Optimal pH and enzyme stability

Observation of optimal enzyme activity was conducted by incubating the sialidase enzymes in pH buffers, including 50 mM sodium acetate (pH 3–6), 50 mM potassium phosphate (pH 7), and 50 mM Tris-HCl (pH 8–10) at 37°C, and observing its activity [20].

### Enzyme-linked lectin assay for sialidase

Enzyme-linked lectin assay was conducted to observe the specific amount of sialic acid remaining from the cells after administering sialidase at numerous concentrations. Chicken erythrocytes in 96-well plates were treated with sialidase for 1 h at 37°C. The cells were washed 3 times with phosphate-buffered saline (PBS, pH 7.3) and fixed immediately with 0.05% glutaraldehyde in PBS. Blocking with 0.2% casein in PBS was performed to stabilize the bound biomolecules and reduce non-specific interactions. Cells were washed once with PBS-0.1% tween 20 (PBST) and incubated at 25°C for 1 h with 20 µg/mL of biotinylated *Sambucus nigra* lectin to detect Neu5Ac α (2,6)-Gal sialic acid and with 20 µg/mL of biotinylated *Maackia amurensis* lectin (GlycoMatrix™, USA) to detect linked Neu5Ac α (2,3)-Gal sialic acid. The cells were washed 4 times in PBST, and secondary detection of the bound lectin was accomplished by incubating the cells with 5 µg of streptavidin-horseradish peroxidase (HRP) (BioLegend^®^, USA) for 1 h at 25°C. The cells were washed 5 times in PBST, followed by the addition of 2,2’-azinobis (3-ethylbenzothiazoline-6-sulfonic acid)-diammonium salt substrate, and then the absorbance was measured at 405 nm. The following e-equation estimated the percentage of remaining sialic acid: 100% × [(absorbance of treated cells–background)/(absorbance of vehicle-treated cells–background)]. The background controls were wells treated with streptavidin-HRP alone without the lectins [21, 22].

### Sialidase toxicity assay

Sialidase toxicity assay was performed using a 10-day-old embryonated chicken egg model (n = 5), and embryo mortality was observed for 72 h by candling every 12 h. The test was performed by diluting the sialidase; subsequently, 0.1 mL of each dilution was inoculated through the chorion-allantoic membrane [23, 24].

### Virus and embryonated chicken eggs

A local isolate of the ND strain chicken/1/78/MSL/19 stored in freeze-dried ampoules was the virus used in this study. Viral propagation was accomplished by injecting intra-allantoic fluid into embryonated chicken eggs and incubating them at 37°C for 2 days. Molecular identification by reverse transcription PCR (RT-PCR) of the fusion protein coding F gene (*Fusion*) in NDV was performed using the following primer sequences: Forward, 5'ATGGGCTCCAGACCTTCTACCA-3' and reverse, 5'CTGCCACTG CTAGTTGTGATAATC3' [24, 25]. The RT-PCR process was carried out using the Master Mix MyTaq™ One-Step RT-PCR Kit (Bioline, Meridian, USA), as per the manufacturer’s instructions.

### Competitive inhibition of embryonated chicken eggs

Observations of the potential for competitive inhibition were divided into five treatment groups: Embryonated chicken eggs injected with a virus, mock control group, and challenge groups. Challenge groups were divided into three administration groups to determine the pre-challenge, competitive, and post-challenge with dilution dose sialidase against NDV infection (n = 5). For the pre-challenge administration, sialidase was given at first, incubated at 37°C for 2 h, and then inoculated with 0.1 mL of 500 embryo infectious dose 50% NDV. In contrast, the post-challenge administration was given in the reverse sequence, that is, by first inoculating the NDV. When sialidase and NDV were simultaneously inoculated into the embryonated chicken eggs, competitive administration was completed to determine if there was any chance of a competitive inhibition process. Embryo mortality was observed twice daily every 12 h for 72 h after injection. Dead embryos were stored at 4°C for further observation. For comparison and evaluation based on the embryo death time, viral titer, and the number of copies of infective viral genomes measured by quantitative RT-PCR (qRT-PCR), embryos that were viable for the first 48 h after inoculation were preserved at 4°C [26, 27].

### Real-time qRT-PCR

Viral RNA from the allantoic fluids was extracted using Viral Nucleic Acid Extraction Kit II Genaid, following the manufacturer’s instructions. Quantus™ Fluorometer (Promega, USA) determined the RNA concentration of each sample. One-step qRT-PCR was conducted using the KAPA SYBR FAST One-Step qRT-PCR Kits (Merck, Darmstadt, Germany), and the fluorescence reading was noted at the end of each cycle, as per the manufacturer’s instructions. The forward and reverse primers according to the F gene of the NDV were as follows: Forward, 5'-TTGATGGCAGGCCTCTTGC-3' and reverse, 5'GGAGGATGTTGGCAGCATT-3' [28]. Serial 10-fold dilutions of standard NDV RNA from 1 to 1 × 10^6^ copies/reaction tube were prepared to construct a standard curve.

### Statistical analysis

The potential for competitive inhibition activity against viral infection on sialic acid receptors in the host by the bacterial sialidase *C. perfringens* was based on several parameters, including embryo mortality, viral titer, and viral copy number. The data were analyzed by one-way analysis of variance and *post hoc* least significant difference to determine the significance of differences among the treatment groups. p ≤ 0.05 was considered statistically significant.

## Results

### *C. perfringens* identification

**Figure-1:**
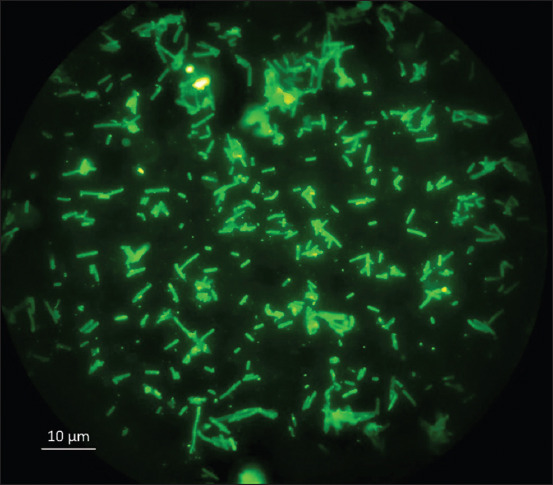
Fluorescence microscopy of *Clostridium* *perfringens* stained with FITC-conjugated anti-*C. perfringens*.

In this study, the PCR identified 11 candidates for *C. perfringens* bacteria isolates, with the identification results based on culture on blood agar, Gram-staining, fluorescent antibody test, and toxinotyping. The results demonstrated that all isolates had the same macroscopic characteristics on blood agar with dual-zone hemolysis around the colony. In addition, microscopic observations with Gram-staining showed Gram-positive bacteria cells, and the fluorescein-5-isothiocyanate (FITC) staining indicated a particular luminescence of *C. perfringens* bacterial cells (Figure-1).

**Figure-2:**
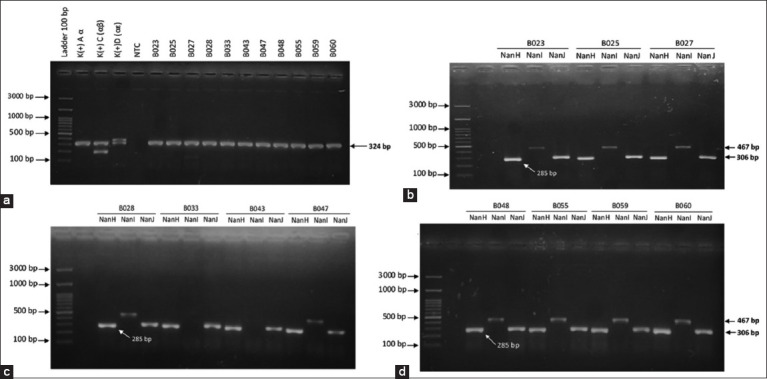
Polymerase chain reaction (PCR) results of amplification of the gene coding for the bacterial toxin *Clostridium perfringens* and the sialidase genes. (a) PCR result on the toxin encoding alpha (cpα), beta (β), and epsilon (cpε). (b-d) PCR results on genes encoding sialidase NanH, NanI, and NanJ in the DNA of *C. perfringens* bacteria.

Based on toxin typing by PCR on the gene coding for toxins alpha (cpα), beta (cpβ), and epsilon (cpε), it indicated that all isolates were *C. perfringens* type A bacteria. The type of bacteria was determined based on the presence of a band at 324 bp on the results of PCR electrophoresis, which indicates the gene coding for alpha-toxin (cp), also known as phospholipase C (Figure-2a). The molecular identification results of the sialidase coding genes demonstrated that almost all *C. perfringens* isolates had *NanH*, *NanI*, and *NanJ* sialidase genes. Of the 11 *C. perfringens*, nine isolates had the *NanH*, *NanI*, and *NanJ* sialidase coding genes (Figure-2b and d), whereas the two other isolates only had *NanH* and *NanJ* sialidase genes (Figure-2c). The result showed variations in the sialidase coding gene from 11 isolates of *C. perfringens* type A bacteria used in this study. However, the PCR results revealed that all isolates carried the sialidase gene and were thought to be capable of producing large amounts of sialidase.

### Synthesis of sialidase and determination of *C. perfringens* isolates

The observations of sialidase activity with the RDE titration method showed that each isolate had various activity values. Overall, three isolates, B025, B048, and B060, had RDE titers of 2^4^; four isolates, B023, B027, B033, and B055, had RDE titers of 2^5^; and two isolates, B043 and B047, had RDE titers of 2^7^. B059 had an RDE titer of 2^6^, and the B028 code isolate had a higher titer than that of the other isolates with 2^8^ dilutions of HI. Bacterial sialidase activity was also measured using the neuraminidase assay kit MAK121 (Merck), yielding a quantitative value in U/L for each crude sialidase from the cultured *C. perfringens* supernatant. These results indicate that *C. perfringens* isolates B028 had an activity of 113.3 U/L or higher, as compared to that of the other *C. perfringens* isolates (Table-2). The isolate can generate a significant amount of sialidase for protein purification and analysis with SDS-PAGE to obtain a sialidase fraction with particular activities.

**Table-2:** Bacteria *C. perfringens* isolate and molecular identification.

Code	Isolation date	Isolate origin	Host	Type	Sialidase gene	RDE	Activity U/L
B023	September 16	Cianjur, West Java	Breeder	A	NanH, NanI, NanJ	25	33,33
B025	September 16	Sukabumi, West Java	Breeder	A	NanH, NanI, NanJ	24	30,00
B027	November 16	Sukabumi, West Java	Layer	A	NanH, NanI, NanJ	25	40,00
B028	February 17	Bogor, West Java	Layer	A	NanH, NanI, NanJ	28	113,33
B033	February 17	Bogor, West Java	Layer	A	NanH, NanJ	25	43,33
B043	January 18	Sukabumi, West Java	Breeder	A	NanH, NanJ	27	66,67
B047	January 19	Cisarua, West Java	Breeder	A	NanH, NanI, NanJ	27	73,33
B048	February 19	Sukabumi, West Java	Layer	A	NanH, NanI, NanJ	24	43,33
B055	June 19	Cianjur, West Java	Breeder	A	NanH, NanI, NanJ	25	46,67
B059	October 19	Sukabumi, West Java	Breeder	A	NanH, NanI, NanJ	26	60,00
B060	December 19	Bogor, West Java	Layer	A	NanH, NanI, NanJ	24	30,00

*C. perfringens=Clostridium perfringens*

### Purification of *C. perfringens* sialidase

Purification was conducted after the production of sialidase from *C. perfringens* B028 bacteria was performed in significant quantities of supernatant culture. It contained a protein concentration of 0.13 mg/mL and a sialidase activity of 0.06 U/mL. The supernatant still contained growth media, so protein precipitation was performed to concentrate the protein from the supernatant, which contained 4.2 mg/mL of protein, with a sialidase activity of 2.1 U/mL. Protein containing the sialidase was then purified by the ion exchange technique to obtain a sialidase fraction with a concentration of 0.4 mg/mL and a sialidase activity of 4.1 U/mL. The final purification stage was conducted by affinity chromatography using oxamic acid agarose to obtain the specific sialidase activity at 75 U/mg.

### Protein analysis

**Figure-3:**
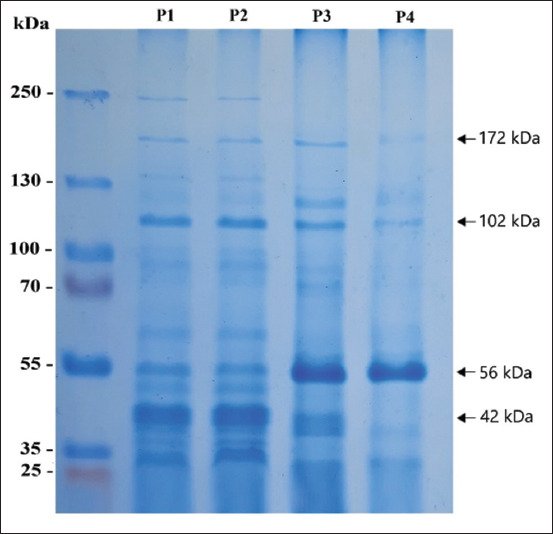
Sodium dodecyl sulfate-polyacrylamide gel electrophoresis (SDS-PAGE) for protein identification of sialidase *Clostridium perfringens* purification step. Observation of protein fraction at each stage of purification using SDS-PAGE. P1 crude sialidase, P2 ammonium sulfate precipitation, P3 ion exchange purification, and P4 affinity chromatography purification.

The SDS-PAGE method was also used to identify the presence of protein to determine sialidase based on molecular weight. The results of SDS-PAGE analysis of the supernatant culture and ammonium sulfate precipitation showed numerous protein bands with varying molecular weights. The ion exchange purification stage showed that numerous bands appearing with molecular weights of 56 kDa and 42 kDa were more dominant. At this stage, 0.4 mg/mL protein was obtained with a sialidase activity of 4.1 U/mL. The purification by affinity chromatography indicated one dominant band at 56 kDa. The purification findings contained a protein concentration of 0.02 mg/mL and a sialidase activity of 1.5 U/mL (Figure-3).

### Optimal pH and sialidase enzyme stability

**Figure-4:**
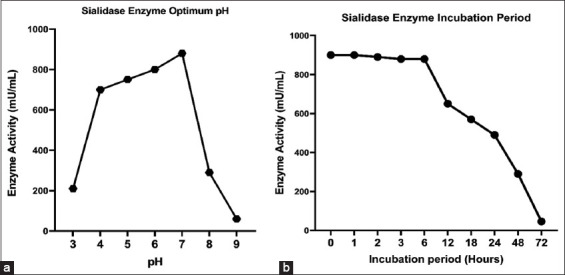
Effect of pH on *Clostridium perfringens* sialidase enzyme activity and stability. Observation of the enzymatic activity was carried out by reacting the enzyme with the substrate so that the U/mL value was obtained. (a) Sialidase enzyme optimum pH, (b) sialidase enzyme incubation period at 37°C.

The optimal pH obtained in this study shows that sialidase is sensitive to an extreme acidic and alkaline pH, so the enzymatic activity decreases dramatically. These results also showed that sialidase at pH 5 and 7 has an optimum activity over the others. Based on the enzyme stability observation, sialidase remained stable after 6 h of incubation and then began to decrease from 880 mU/mL to 650 mU/mL after 12 h of incubation (Figure-4).

### Sialidase toxicity assay in ovo

Toxicity assay observations neither showed embryo mortality up to 72 h after injection nor did any significant embryo abnormalities occur in the placebo or treatment groups.

### Enzyme-linked lectin assay for sialidase

**Figure-5:**
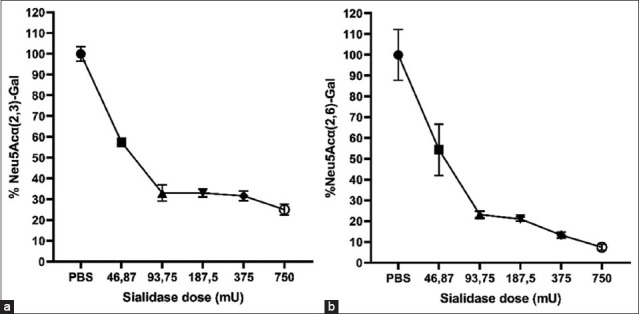
Sialic acid removal on the surface of chicken red blood cells after treatment by *Clostridium perfringens*. (a) Observation of sialic acid Neu5Ac (2,3)-Gal Neu5Ac and (b) (2,6)-Gal remaining after treatment with sialidase with enzyme-linked lectin assay method.

The enzyme lectin assay against sialic acid receptors using chicken RBC revealed a decrease in sialic acid levels compared to that of the controls in Neu5Ac (2,3)-Gal and Neu5Ac (2,6)-Gal after several doses of sialidase administration. Sialic acid levels began to decrease after the administration of 46.87 mU sialidase, subsequently decreasing to 54.4 ± 12% (Neu5Ac a [2,6] gal) and 57.5 ± 2% (Neu5Ac a [2,3] gal). The sialic acid decreased greatly at the highest dose of 750 mU sialidase so that the remaining sialic acid was 7.46 ± 1.4% (Neu5Ac [2,6]-Gal) and 25.07 ± 2.6% (Neu5Ac [2,3]-Gal). Following these findings, sialidase derived from *C. perfringens* tends to hydrolyze the sialic acid receptor Neu5Ac (2,6)-Gal higher than Neu5Ac (2,3)-Gal in chicken erythrocytes (Figure-5).

### Competitive inhibition in the *in ovo* model

Observations in the mock control group showed no hemagglutination (HA) titer of NDV, whereas in the NDV treatment control, the HA titer was 7 log2 or 128. In addition to the HA titer, embryonic death was observed in the mock control; no death occurred up to 72 h, whereas in the NDV control group, embryonic death was observed around 40.8 ± 6.57 h post-inoculation. Based on the HA titer in the pre-challenge administration group, multiple doses demonstrated a significant difference (p < 0.05) with the NDV treatment control. At doses of 375, 187.5, and 93.75 mU sialidase, allantoic fluid HA titers were obtained, with a mean of 64 ± 0, 70.4 ± 35.1, and 57.6 ± 14.3, respectively. These findings show that there is interference with NDV replication in embryonated chicken eggs due to the presence of sialidase.

**Figure-6:**
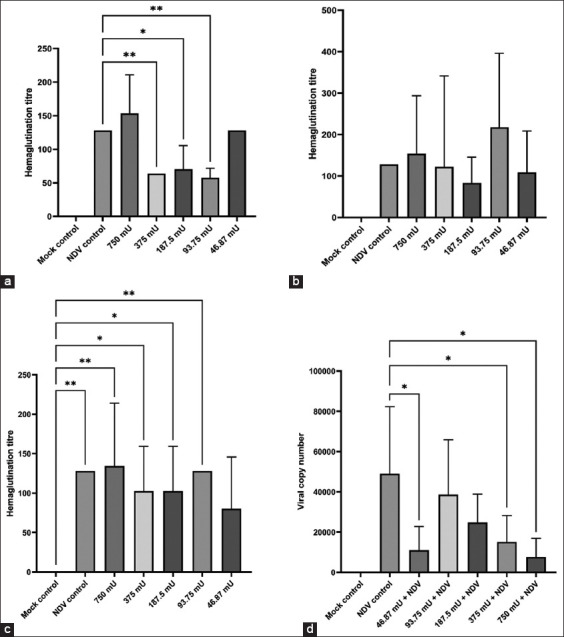
Observation of hemagglutination titer and measurement of viral copy gene by quantitative reverse transcription polymerase chain reaction (qRT-PCR). (a) Mean virus titer on embryonated chicken eggs in pre-challenge administration groups. (b) Mean virus titer on embryonated chicken eggs in competitive administration groups. (c) Mean virus titer on embryonated chicken eggs in post-challenge administration groups. (d) Observation of viral copy number by qRT-PCR of allantoic fluid results in the pre-challenge administration groups.

Furthermore, interference with viral replication based on the HA titer in the allantoic fluid was observed at sialidase doses ranging from 375 mU to 93.75 mU. In contrast, at a dose of 46.87 mU, there was an increase in the HA viral titer, with a mean of 128 ± 0 (Figure-6). However, administering several doses of sialidase in the competitive and post-challenge administration groups showed no significant difference. In addition, embryonic death showed no significant difference between the groups. Interestingly, the competitive administration of 93.75 mU of sialidase showed higher HA titers than that in the NDV treatment control.

To determine viral replication interference, qRT-PCR was used to quantify the amount of virus based on the number of copies of the infective viral gene in the pre-challenge administration group. A standard curve was also established, with detection ranging from 1 to ×10^6^ copies/reaction (R^2^ = 0.993). The findings demonstrated a significant difference in the viral copy numbers of allantoic fluid (p < 0.05) at treatment doses of 750 mU, 375 mU, and 46.87 mU sialidase against the NDV control group based on the viral copy numbers (Figure-6). This indicates an inhibition of the F gene replication from NDV at the dose of sialidase.

## Discussion

The study explained the ability of purified sialidase from *C. perfringens* to inhibit viral replication in cells by hydrolyzing sialic acid from the surface of host cells and interfering with viral multiplication in allantoic fluid from embryonated eggs. Following this study’s molecular analysis of the toxin gene, all isolates solely contained the alpha-toxin encoding gene. The selection of *C. perfringens* type A in this study prevented pathogenic potential extracellular toxins, which are frequently encoded in accessory genomes such as b, and e toxins are usually encoded on plasmids carried on mobile genetic elements, most commonly on large conjugative plasmids [29, 30]. Consistent with the research on *C. perfringens* genomes, these genes are associated with strain-to-strain variation in virulence and phenotypic characteristics [31].

Molecular identification of the sialidase genes in this study showed that eight isolates had all three of these genes, whereas the other two isolates had only *NanH* and *NanJ*. Genome sequencing has been shown and recognized *C. perfringens* strains that commonly have three sialidase coding genes, namely, *NanH* (non-secreted, 43 kDa), *NanI* (secreted, 77 kDa), and *NanJ* (secreted, 122 kDa), located in the conserved chromosomal region [32–34]. Unlike *NanH*, which only has a catalytic domain, *NanI* and *NanJ* have additional carbohydrate-binding modules that can increase the binding affinity to polyvalent substrates [34, 35]. Although almost all the isolates had all three sialidase genes molecularly in this study, the enzyme activity produced differed between isolates. This may be influenced by regulatory genes that play a role in sialidase synthesis. Sialidase, related to ReeS (Regulator of extracellular enzymes Sensor), encodes a sensor histidine kinase that regulates extracellular production [5, 36, 37].

Purifying the sialidase enzyme in this study also demonstrated many shortcomings, including the decrease in enzyme activity (activity recovery) to obtain sialidase. However, based on the results of SDS-PAGE and specific enzymatic activity, it showed high purity, with an activity of 1.5 U/mL for treating embryonated chicken eggs. Therefore, in this study, we discovered a sialidase with a molecular weight of 56 kDa based on the SDS-PAGE analysis. The molecular weight was determined based on the sialidase activity of the protein fraction analyzed. Thus, the molecular weight corresponds to the commercial sialidase neuraminidase (Sialidase, from *C. perfringens* 11585886001 Roche), at around 60 kDa.

In the previous study conducted by Wang [38] regarding the pH stability of *C. perfringens*, all three sialidase enzymes showed optimal activity at pH 5.5. However, in this study, the activity of the sialidase enzyme at pH 5, 6, and 7 did not appear drastically different. A reduction of the activity of the enzyme sialidase at pH 9 was found, which indicates the sensitivity of this enzyme to alkaline conditions [35, 36] (Figure-4).

This study’s embryonated chicken egg toxicity assay showed that it did not cause embryo death or abnormalities up to 72 h after injection. In addition, this study showed no difference between the placebo and treatment groups at several doses of *C. perfringens* sialidase. Many non-pathogenic bacteria also secrete sialidases to scavenge host cell sialic acid for use as a carbon and energy source [39, 40]. In addition, sialic acid-binding adhesins frequently facilitate pathogen and toxin associations with the host mucosa. However, no pathogenic role for these enzymes that remove terminal sialic acid residues from glycoproteins and glycolipids has yet been identified [33, 40, 41].

Sialidase cleaves terminal sialic acid residues that are alpha-linked to oligosaccharide chains found on proteins and lipids. The enzyme-linked lectin assay using chicken erythrocytes in this study also proved that sialidase from *C. perfringens* can hydrolyze α-2,3 and α-2,6 sialic acid receptors on the cell surface. Chicken erythrocytes were used because α-2,3 sialic acid was found at a higher amount than that of α-2,6 sialic acid, although both are present in these cells [42]. Surprisingly, this study’s hydrolytic activity of sialidase appeared higher against α-2,6 sialic acid receptors than α-2,3 sialic acid. The previous study conducted by Li and McClane [35] has shown that there is an affinity of each *NanH*, *NanI*, and *NanJ* *C. perfringens* sialidase to certain sialic acid receptors; however, these sialidases can hydrolyze all three α-2,3, α-2,6, or even α-2.8 sialic acid receptor linkages [35].

In this study, the competitive inhibition *in ovo* model revealed that *C. perfringens* sialidase interfered with viral replication, as evidenced by a decrease in HA titer in the pre-challenge administration group compared with that in the NDV controls. This can be observed on the basis of the significant difference in titers from the pre-challenge administration of *C. perfringens* sialidase compared to that in the NDV control treatment. While in competitive and post-challenge administration, there was no significant difference between the NDV treatment controls. The competitive administration showed increasing HA titers of NDV in the allantoic fluid. This may be related to the mechanism of NDV infection and the interaction between the sialic acid receptor and the sialidase enzyme administered simultaneously. NDV infections are initiated when virus particles bind to sialic acid receptor molecules on the surface of target cells that are mediated by HN proteins [43]. Unlike influenza viruses, which undergo endocytosis, NDV fusion occurs at the cell surface. Recognition of sialic acid-containing receptors by HN somehow leads to a conformational change in F to its fusogenic state [44]. Completing this process allows virion contents, including ribonucleoproteins, to enter the cytoplasm of target cells [43].

The observations of infective virus gene copies by qRT-PCR against RNA in allantoic fluid showed a significant difference, with a decrease in the number of allantoic fluid viral copies against the NDV control group. This indicates an inhibition of the *F* gene replication from NDV at the dose of sialidase. The number of copies of the *F* gene indicates the amount of NDV in the allantoic fluid. The *F* gene plays an important role in the initial infection along with HN, two surface glycoproteins in NDV. Alternatively, the F protein directs the membrane fusion between the viral envelope and the cell membrane. It is a fundamental aspect that plays a key role in viral virulence and tissue tropism [45]. The previous studies [22, 46] have shown that the presence of sialic acid on the cell surface of a respiratory epithelial cell can increase the efficiency of viral infection. In contrast, when sialic acid on the cell surface is removed by sialidase, it reduces viral binding to host cells.

However, incomplete enzymatic desialylation to eliminate sialic acid may lead to viral infection from residual sialic acid on the cell surface. This research follows previous studies [11, 12] that revealed the possibility of bacterial sialidase, which functions to remove sialic acid from the surface of epithelial cells. So that mechanism can prevent attachment and subsequent infection by respiratory viruses that use sialic acid as a receptor to perform the initial step of endocytosis. The failure of viral endocytosis caused by sialidase administration results in a decrease or even prevention of virus entry into cells due to a reduction or loss of sialic acid receptors on the surface of host cells. This causes interference with viral replication and translation, so the process of multiplying ND viruses in cells is disrupted.

## Conclusion

This study has revealed the potency of sialidase obtained from *C. perfringens* as a sialic acid receptor competitive inhibitor against NDV *in ovo*. As presented in this study, the purified sialidase from *C. perfringens* could interfere with NDV multiplication by reducing viral titer and copy number in allantoic fluids without significantly affecting the toxicity of chicken embryos at different concentrations. However, it is too hard to determine the appropriate dose of the sialidase target receptor in *in ov*o model, considering the sialic acid receptor on the surface of embryo cells are very complex compared with the epithelial surface. Therefore, further research by monolayer cells *in vitro* and *in vivo* investigations is needed to validate the efficacy of this substance for the prophylactic treatment of infectious viral disease.

## Authors’ Contributions

RSK: Conceptualization and design of the study. ST, FI, and PPS: Supervision. RSK and CMHN: Data acquisition, analysis, and interpretation. OSMS and LN: Drafted and revised the manuscript. All authors have read and approved the final manuscript.
